# Supplementary material for: The role of resource transfer in positive, non-additive litter decomposition
Source: PLoS One. 2019 Nov 18;14(11):e0225337. doi: 10.1371/journal.pone.0225337 (PMC6860423; doi:10.1371/journal.pone.0225337)
Supplement: S1 Appendix — (DOCX) [file pone.0225337.s001.docx]

The role of mineral nutrient and labile carbon transfers between litter types in positive, non-additive decomposition

Na Yin and Roger T. Koide^*^

Department of Biology, Brigham Young University, Provo, UT 84602, USA

^*^Corresponding author

rogerkoide@byu.edu (Roger T. Koide)

**Measurement of cellobiohydrolase (CBH, EC 3.2.1.91) activity**

For each replicate, approximately 0.5 g (fresh weight) ground litter was added to 50 mL distilled water in a capped 50 mL centrifuge tube and homogenized by hand for 30 s. Because enzyme activity is pH sensitive, we used water rather than buffer so the solution remained at the pH of the litter. Then, 1 mL of litter homogenate was immediately transferred to a 2 mL microfuge tube. The ends of the pipette tips were clipped to accommodate litter particles. The MUB-CB (0.5mL of 200μM), was added to the homogenates and the microfuge tubes were placed horizontally on a mixer at low speed for 45 min incubation. Upon completion of the incubation, 0.5 mL of 50 mM sodium hydroxide (NaOH) was added to stop the reaction, bringing the total volume in each tube to 2 mL. Tubes were then centrifuged at 10,000 x g for 1 min. A 200 μL aliquot of supernatant from each sample was added to each of 8 wells of a black, polystyrene 96-well microplate to yield eight analytical replicates per experimental replicate. Four additional columns (each consisting of 8 replicate wells) were filled in the following order: MUB standard wells contained 100 μL water, 50 μL of 10 μM β-methylumbelliferone (MUB, Sigma-Aldrich) and 50 μL of 50 mM NaOH. The substrate blank wells contained 100 μL water, 50 μL of 200 μM MUB-CB and 50 μL of 50 mM NaOH. The sample autofluorescence blank wells contained 50 μL water, 100 μL of sample supernatant and 50 μL of 50 mM NaOH. The quenching control wells contained 100 μL of sample supernatant, 50 μL of 10 μM MUB and 50 μL of 50 mM NaOH. Fluorescence was determined using a Biotek Synergy HT spectrophotometer with a 360 nm excitation filter and 460 nm emission filter. CBH activities were calculated from average fluorescences of the eight analytical replicates.

**Table A. Results of one-way analysis of variance (ANOVA) for oat straw decomposition rate determined after 25 d incubation in experiment 2.**

| Source of variation | df | SS | MS | *F* | *P* |
| --- | --- | --- | --- | --- | --- |
| Treatment | 3 | 56.0 | 18.7 | 12.7 | **0.0002** |
| Residuals | 16 | 23.5 | 1.47 |  |  |
| Total | 19 | 79.5 |  |  |  |

**Table B. Results of one-way analysis of variance (ANOVA) for cellobiohydrolase activity in oat straw measured after 1 week of incubation in experiment 3.1.**

| Source of variation | df | SS | MS | *F* | *P* |
| --- | --- | --- | --- | --- | --- |
| Treatment | 6 | 4.65x10^5^ | 7.75 x10^4^ | 3.19 | **0.0133** |
| Residuals | 35 | 8.51x10^5^ | 2.43 x10^4^ |  |  |
| Total | 41 | 1.32 x10^6^ |  |  |  |

**Table C. Results of one-way analyses of variance (ANOVA) for oat straw decomposition rate at 14, 21 and 28 d of incubation in experiment 3.2.**

| Incubation time | Source of variation | df | SS | MS | *F* | *P* |
| --- | --- | --- | --- | --- | --- | --- |
| 14 d | Treatment | 3 | 4.67 | 1.56 | 0.218 | 0.883 |
|  | Residuals | 27 | 192 | 7.13 |  |  |
|  | Total | 30 | 197 |  |  |  |
|  |  |  |  |  |  |  |
| 21 d | Treatment | 3 | 33.0 | 11.0 | 2.93 | 0.0511 |
|  | Residuals | 28 | 105 | 3.76 |  |  |
|  | Total | 31 | 138 |  |  |  |
|  |  |  |  |  |  |  |
| 28 d | Treatment | 3 | 130 | 43.4 | 14.4 | **<.0001** |
|  | Residuals | 28 | 84.3 | 3.01 |  |  |
|  | Total | 31 | 214 |  |  |  |

**Table D. Results of one-way analyses of variance (ANOVA) for cellobiohydrolase activity in oat straw at 14, 21 and 28 d of incubation in experiment 3.2.**

| Incubation time | Source of variation | df | SS | MS | *F* | *F* |
| --- | --- | --- | --- | --- | --- | --- |
| 14 d | Treatment | 3 | 6.61x10^6^ | 2.20x10^6^ | 48.7 | **<0.0001** |
|  | Residuals | 28 | 1.27x10^6^ | 4.53x10^4^ |  |  |
|  | Total | 31 | 7.88x10^6^ |  |  |  |
|  |  |  |  |  |  |  |
| 21 d | Treatment | 3 | 4.17x10^-6^ | 1.39x10^-6^ | 45.4 | **<0.0001** |
|  | Residuals | 28 | 8.56x10^-7^ | 3.06x10^-8^ |  |  |
|  | Total | 31 | 5.02x10^-6^ |  |  |  |
|  |  |  |  |  |  |  |
| 28 d | Treatment | 3 | 2.38 | 0.794 | 54.2 | **<0.0001** |
|  | Residuals | 28 | 0.389 | 1.39x10^-2^ |  |  |
|  | Total | 31 | 2.77 |  |  |  |

*Notes:* For 21 d samples, statistical analyses were performed on data that were reciprocally transformed; for 28 d samples, statistical analyses were performed on data that were log transformed.

**Table E. Results of one-way analysis of variance (ANOVA) for oat straw decomposition rate determined after 28 d incubation in experiment 4.**

| Source of variation | df | SS | MS | *F* | *P* |
| --- | --- | --- | --- | --- | --- |
| Treatment | 3 | 53.5 | 17.8 | 4.87 | **0.0075** |
| Residuals | 28 | 102 | 3.66 |  |  |
| Total | 31 | 156 |  |  |  |

**Table F. Results of one-way analysis of variance (ANOVA) for cellobiohydrolase activity determined after 28 d incubation in oat straw in experiment 4.**

| Source of variation | df | SS | MS | *F* | *P* |
| --- | --- | --- | --- | --- | --- |
| Treatment | 3 | 2.92x10^7^ | 9.74x10^6^ | 47.5 | **<0.0001** |
| Residuals | 28 | 5.74x10^6^ | 2.05x10^5^ |  |  |
| Total | 31 | 3.50x10^7^ |  |  |  |

**Table G. Results of one-way analysis of variance (ANOVA) for mean oat straw decomposition rate determined after 28 d incubation in experiment 5.**

| Source of variation | df | SS | MS | *F* | *P* |
| --- | --- | --- | --- | --- | --- |
| Treatment | 2 | 64.5 | 32.2 | 12.6 | **0.0003** |
| Residuals | 21 | 53.9 | 2.56 |  |  |
| Total | 23 | 118 |  |  |  |

**Table H. Results of one-way analysis of variance (ANOVA) for cellobiohydrolase activity in oat straw determined after 28 d incubation in experiment 5.**

| Source of variation | df | SS | MS | *F* | *P* |
| --- | --- | --- | --- | --- | --- |
| Treatment | 2 | 0.801 | 0.401 | 50.6 | **<0.0001** |
| Residuals | 21 | 0.166 | 7.93x10^-3^ |  |  |
| Total | 23 | 0.967 |  |  |  |

*Notes:* Statistical analyses were performed on data that were log transformed.

**Table I. Results of one-way analysis of variance (ANOVA) for oat straw decomposition rate determined after 28 d incubation in experiment 6.**

| Source of variation | df | SS | MS | *F* | *P* |
| --- | --- | --- | --- | --- | --- |
| Treatment | 3 | 0.110 | 0.0368 | 3.55 | **0.0330** |
| Residuals | 20 | 0.207 | 0.0104 |  |  |
| Total | 23 | 0.318 |  |  |  |

*Notes:* Statistical analyses were performed on data that were log transformed.

**Table J. Results of one-way analysis of variance (ANOVA) for cellobiohydrolase activity in oat straw determined after 28 d incubation in experiment 6.**

| Source of variation | df | SS | MS | *F* | *P* |
| --- | --- | --- | --- | --- | --- |
| Treatment | 3 | 0.945 | 0.315 | 20.1 | **<0.0001** |
| Residuals | 19 | 0.298 | 0.0157 |  |  |
| Total | 22 | 1.24 |  |  |  |

*Notes:* Statistical analyses were performed on data that were log transformed.

**Table K. Results of one-way analysis of variance (ANOVA) for oat straw decomposition rate after 28 d incubation in experiment 7.**

| Source of variation | df | SS | MS | *F* | *P* |
| --- | --- | --- | --- | --- | --- |
| Treatment | 3 | 27.3 | 9.11 | 9.52 | **0.0002** |
| Residuals | 28 | 26.8 | 0.957 |  |  |
| Total | 31 | 54.1 |  |  |  |

**Table L. Results of one-way analysis of variance (ANOVA) for oat straw decomposition rate after 28 d incubation in experiment 8.**

| Source of variation | df | SS | MS | *F* | *P* |
| --- | --- | --- | --- | --- | --- |
| Treatment | 3 | 24.6 | 8.20 | 5.58 | **0.0039** |
| Residuals | 28 | 41.1 | 1.47 |  |  |
| Total | 31 | 65.7 |  |  |  |
