# Supplementary material for: The role of resource transfer in positive, non-additive litter decomposition
Source: PLoS One. 2019 Nov 18;14(11):e0225337. doi: 10.1371/journal.pone.0225337 (PMC6860423; doi:10.1371/journal.pone.0225337)
Supplement: Attachment — Submitted filename: response to reviewers.docx [file pone.0225337.s003.docx]

Professor Shuijin Hu

Academic Editor

30 October 2019

Dear Shuijin,

Thank you very much for handling our manuscript “The role of resource transfer in positive, non-additive litter decomposition”.

We have revised the manuscript in accordance with PLOS ONE style templates. Moreover, we have responded to the following reviewer comments in the following ways:

Reviewer #1: Major comments:
1. I suggest the authors to give clear hypotheses they would like to test in the Introduction;
We have added explicit hypotheses.

2. The major concern I have is that all the experiments can only explain the effect of water, N, C, other mineral nutrients or various combinations of these on oat decomposition, rather than the positive effect of clover litter on oat decomposition, which the authors want to address;

In fact, we did address the positive effect of clover litter on oat decomposition. We had several hypotheses to explain the clover effect, including its contribution to oat straw of water, N, C and other mineral nutrients.

3. How the authors exclude the role of microbes from clover litter in stimulating oat straw decomposition.

We have explained that in a previously published paper, the positive net effect of clover litter on oat straw decomposition was correlated with increased microbial activity. In this particular manuscript, we have extended our previous finding to the mechanisms responsible for the microbial effect.

Specific Comments:
• Line 46, please define C, where it first appeared in the Text

This has been added.

• Line 85, more references should be provided

There are already 3 references for this single point. We do not feel it is necessary to add more to make the point.

• Line 291, “addition” typo

This has been corrected.

• Line 459-471, no studies were cited

We have added a citation here.

Reviewer #2: Please see the attached document to view my editing suggestions. The manuscript is well-written and covers an important topic. The authors did a fine job in testing multiple treatments to better understand the effects of clover residues on oat straw decomposition. My primary editing suggesting is that the authors spend more time discussing the practical implications of their findings. Clover and oat are common cover crops and often used in mixtures. Findings from this study have practical implications for N management on subsequent crops.

We have altered the manuscript as suggested by this reviewer in the manuscript.

However, we have not added further discussion on practical implications for N management on subsequent crops because the context of this work is larger than N management in agricultural systems. For example, the work has implications with respect to the cycling of mineral nutrients, the fluxes of C into the atmosphere, and the transformation of relatively labile litter into stable soil organic matter. We have added these considerations into the discussion.

With best wishes,

Roger T. Koide

Professor of Biology
